# Revolution in UK General Practice Due to COVID-19 Pandemic: A Cross-Sectional Survey

**DOI:** 10.7759/cureus.9573

**Published:** 2020-08-05

**Authors:** Sanjeev C Sharma, Sonita Sharma, Arjuna Thakker, Gopal Sharma, Mohamed Roshan, Vivek Varakantam

**Affiliations:** 1 Family Medicine, Whittington Health NHS Trust, London, GBR; 2 Family Medicine, University of Buckingham Medical School, Buckingham, GBR; 3 Family Medicine, University Hospitals of Leicester NHS Trust, Leicester, GBR; 4 Family Medicine, Fosse Medical Centre, Leicester, GBR; 5 Family Medicine, Willows Health, Leicester, GBR; 6 Family Medicine, Croft Medical Centre, Leicester, GBR

**Keywords:** covid-19, corona virus, general practice, survey research, coronavirus pandemic, primary care

## Abstract

Objectives

To assess how UK General Practitioners (GPs) and Practice Managers (PMs) have coped with the challenges posed by the coronavirus disease-19 (COVID-19) pandemic and whether they felt adequately supported by the wider National Health Service (NHS).

Methods

This is a cross-sectional survey. All GPs and PMs (total 1,354) in Leicester, Leicestershire, and Rutland (LLR) were invited to participate in an online questionnaire.

Results

A total of 95 invitees completed the survey. Over a quarter had required time off work due to COVID symptoms or contact. All respondents described either introducing or increasing the use of remote patient consultations. Most striking was the rise in video consultations from just 3% to 95% during the pandemic. Almost half of the feedback on the usefulness of remote consultations were positive, 16% were negative and 17% were mixed. The most commonly cited benefit was time efficiency. Drawbacks of remote consultations included technical difficulties and poor patient communication. Practice premises, systems and processes also required significant modifications during the pandemic to ensure the provision of safe clinical care, including reception screens, one-way patient flow, greater infection prevention measures. However, despite their ability to introduce such widescale change virtually overnight, over 10% of respondents reported that the strain had placed their practice at risk of closure. Over half of respondents felt they were not provided with adequate personal protective equipment (PPE) for the safety of their staff. Perception of the support provided by NHS England and the Clinical Commissioning Groups (CCGs) was rather mixed, although additional guidelines were broadly welcomed. The most requested enduring changes related to remote patient consultations (59%) and remote triage (19%). However, in order to support such largescale permanent change, study respondents felt that a different funding and financial structure is required together with improved IT infrastructure, greater patient education and a more supportive regulatory environment.

Conclusions

COVID-19 has substantially accelerated the pace of change within NHS primary care. The long-term fear is that there may be insufficient financial and clinical backing from regulatory bodies to support such rapid and far-reaching changes.

## Introduction

The coronavirus disease-19 (COVID-19) pandemic prompted an unprecedented restructuring and rapid adaptation of all health and social care services [1]. Delivery of primary care services is complex and has been gradually evolving since the introduction of the National Health Service (NHS) Five Year Forward View in 2014 [2] and primary care networks (PCNs) in 2019 [1,3]. However, such evolutionary change was triggered into an overnight revolution by the recent pandemic. Numerous changes were introduced such as increased usage of telemedicine [4] and closer working with NHS England and Public Health England (PHE), e.g. for personal protective equipment (PPE) guidance [5].

This cross-sectional survey aims to assess how General Practitioners (GPs) and Practice Managers (PMs) have coped with the challenges posed by the COVID-19 pandemic and whether they felt adequately supported by the wider NHS.

## Materials and methods

Participants and procedures

All GPs and PMs in Leicester, Leicestershire, and Rutland (LLR) were invited to participate in an online cross-sectional survey (supplementary figures in the Appendices) designed using Google Forms. Emails were sent to a potential 1,354 participants (1,111 GPs, 243 PMs). Responses were kept anonymous to facilitate honest feedback. Data was collected between May 26, 2020 to June 14, 2020. This was a voluntary survey of healthcare professionals, so no ethical approval was required.

Online survey

The 28-question survey included a range of open and closed questions and was developed collaboratively by the authors SCS and SMS, whilst GKS, MR, and VV evaluated the questionnaire and provided amendments. The responses were either checkbox, Likert scale (0 to 10) or long answer. 

Questions were designed to cover a range of domains namely: 1. Respondent demographic information, 2. Changes to consultation methods, 3. Modifications to premises, 4. Modifications to practice systems and processes, 5. Usefulness of support from governing bodies (NHS England, PHE, and Clinical Commissioning Groups (CCG)), 6. Benefits of implemented changes.

The survey was tested by a GP and a PM to establish face and content validity, who also provided feedback on ease of completion (estimated completion time 5-10 minutes). The final version was confirmed by all authors before wider dissemination.

Data analysis

Quantitative statistical analysis was performed using IBM Statistical Packing for Social Sciences (SPSS) software, version 25.0 (SPSS, Chicago, IL, USA). The qualitative data collected from the five open-ended survey questions were analysed by SCS and SMS independently to reduce bias. They initially identified common themes and then subsequently coded the responses independently. The researchers convened to discuss discrepancies. Frequencies of common themes were then recorded.

## Results

Respondent demographic information

Ninety-five out of 1,354 invitees completed the online survey (7% response rate). All respondents reported to be working during the COVID-19 pandemic. 10% were working completely from home while just over a third did no remote working. A significant proportion of the respondents were GP partners (46.3%) and over 86% had more than 10 years of NHS experience. Table 1 details the full description of respondent demographic information. Over a quarter (28.4%) had required time off work due to COVID symptoms or contact.

**Table 1:** Respondent demographic information

Demographic Information	Number of Respondents (%)
Job Title	
GP Partner	44 (46.3%)
Salaried GP	14 (14.7%)
Locum GP	5 (5.3%)
Practice Manager	32 (33.7%)
Number of Years NHS Experience	
< 5 years	3 (3.2%)
5 – 10 years	10 (10.5%)
> 10 years	82 (86.3%)
Black, Asian and Ethnic Minority (BAME) Group Member	
Yes	52 (54.7%)
No	43 (45.3%)
Practicing in the City vs County	
City	50 (52.6%)
County	45 (47.4%)

Changes in patient consultation methods

**Figure 1:**
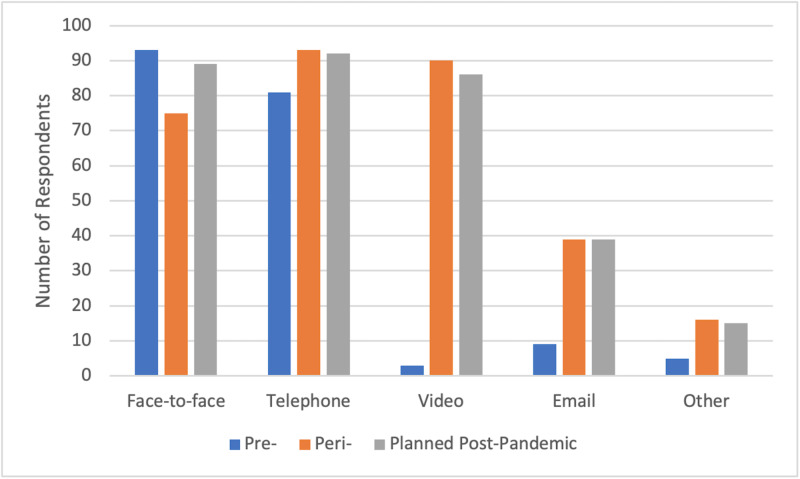
Forms of patient consultation used pre-, peri- and planned post-pandemic

All respondents described either introducing or increasing the use of remote patient consultations. 81 of the 95 participants reported an increase in teleconsultations of 80-100% compared to pre-COVID levels. Figure 1 depicts the reported usage of different modes of patient consultation before, during and anticipated after the pandemic. Most striking was the rise in video consultations from just 3% to 95% during the pandemic and almost all respondents were also planning to continue using this technology (91%). In addition, there were significant increases in email and telephone consultations.

**Figure 2:**
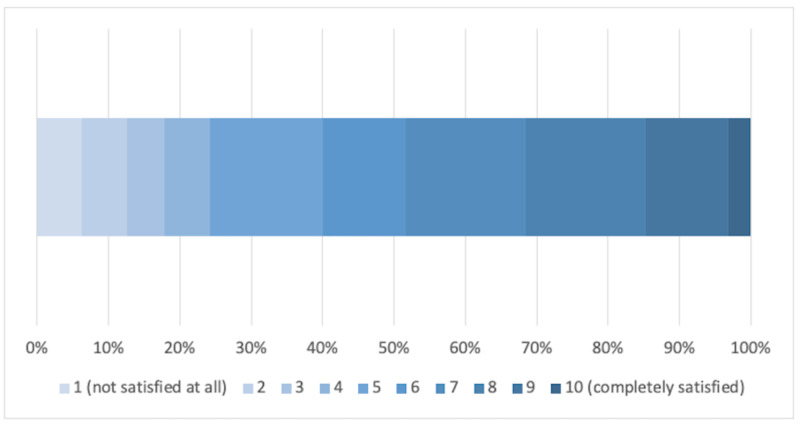
Satisfaction with IT support and guidance for remote healthcare

As illustrated in Figure 2, satisfaction with the support provided on the use of remote technology was rather mixed but overall acceptable given that this was an unprecedented emergency requiring urgent action at all tiers of the NHS. 23 out of the 95 respondents described the support as insufficient (< 5/10) while over 75% were content with the guidance provided (≥ 5/10).

Almost half of the GP/PM feedback on the utility of remote patient consultations were positive, 16% were negative and 17% were mixed, whilst 18% failed to respond. The most commonly cited benefit was time efficiency (17%). 13% reported particular value in certain circumstances, such as monitoring care home patients and certifying deaths and 7% described the benefits of remote triage. Other obvious suggestions included that remote encounters were safer than face-to-face consultations (3%) and also more flexible/convenient (3%). 

Conversely, the drawbacks of remote consultations included technical difficulties (10%) and poor patient communication (7%). GPs cited problems detecting non-verbal cues and language barrier issues. 6% described remote consultations as actually being time inefficient, including doubling the workload when there was need to progress to a face-to-face encounter. One doctor explained how patients were less considerate towards his/her time pressures in the absence of a busy physical waiting room; 3% felt that the video and photo technology for visualising skin lesions was of poor quality, although one GP reported the converse; 3% felt that remote consultations were potentially less safe than actually seeing a patient and one clinician highlighted the need to provide enhanced safety netting advice. Two respondents also reported concern over burn out as they found teleconsultations consultations more tiring than face-to-face meetings. 

Respondents were also asked to provide any patient feedback they may have received on their increased usage of remote consultations; 56% described positive responses, 17% reported mixed feedback, whilst only 2% had received negative patient comments. Patients had highlighted various advantages such as improved access and reduced waiting time (14%), increased convenience of consulting from home (13%), and time efficiency (5%). The main drawbacks for patients were technical difficulties (10%), particularly for elderly populations, and 4% found teleconsultations impersonal compared to face-to-face meetings with their doctor. However, only 8% of respondents stated that their patients had expressed an actual preference for face-to-face consultations. 

Changes to practice premises

The study revealed that practices had to make significant changes to their premises in order to continue the provision of safe services during the COVID-19 pandemic. The most commonly reported changes were reducing the number of waiting room chairs to maintain social distancing, additional cleaning, introducing a reception screen, creating separate entrance and exit doors. See Table 2 for the full breakdown on premise modifications.

**Table 2:** Modifications to premises

Modifications to Premises	Number of Respondents (%)
Reception screen	74 (77.9%)
Separate entrance and exit doors	53 (55.8%)
Fewer waiting room chairs	82 (86.3%)
Automated doors	19 (20%)
Automated hand driers	14 (14.7%)
Additional cleaning	75 (78.9%)
Increased signage	4 (4.2%)
Hot and cold zones	7 (7.4%)
Other (video-controlled entry, security showing patients to don and doff, reduced clutter, one-way system)	5 (5.3%)

Changes to practice systems and processes

Table 3 shows that significant changes were also made to practice systems and processes, such as introducing staff risk stratification policies.

**Table 3:** Changes to systems and processes

Changes to Systems/Processes	Number of Respondents (%)
Longer consultation times	47 (49.5%)
Cleaning gaps between patients	68 (71.6%)
Staggered surgeries to avoid overcrowding	60 (63.2%)
Patient masks	55 (57.9%)
Hand sanitisers at entrance	72 (75.8%)
Revised practice website/patient information leaflets	80 (84.2%)
Greater use of patient text messaging	84 (88.4%)
Staff risk stratification	86 (90.5%)
Greater offsite working	80 (84.2%)
Additional staff training	64 (67.4%)
Revised policies and procedures	86 (90.5%)
Greater administrative burden (e.g. daily reporting)	75 (78.9%)
More frequent team meetings	57 (60.0%)
Remote team meetings	76 (80.0%)
Additional financial burden	67 (70.5%
Closer liaison with neighbouring practices	34 (35.8%)

11.6% of participants indicated that their practice had been at risk of closure at some point due to the strains of the pandemic, while 8.4% reported they were unsure of this. 83.2% felt that they had been given sufficient autonomy to tailor their services to fulfil the needs of their own patients, staff and premises.

Support from governing bodies

**Figure 3:**
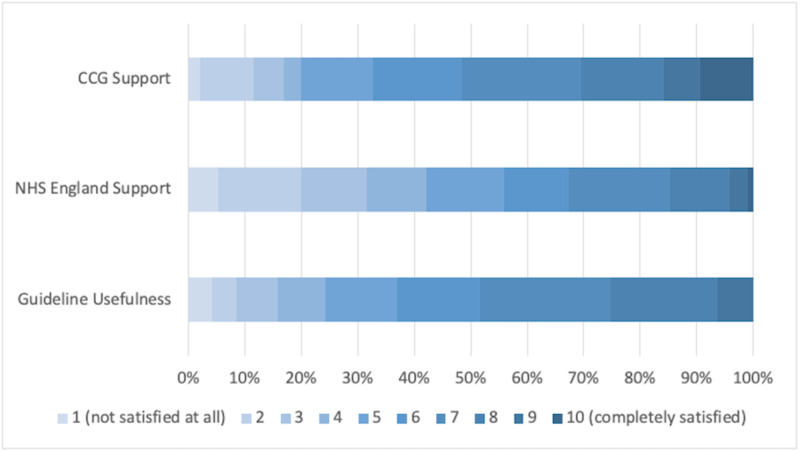
Support by Clinical Commissioning Groups, NHS England and usefulness of guidelines

Figure 3 demonstrates that NHS England was perceived to be the least useful by study respondents (57.9% scoring ≥ 5/10). Similarly, over half (56.8%) of respondents highlighted that they had not received adequate PPE. In contrast, local CCG support was described as the most helpful intervention (80.0% scoring ≥ 5/10), whilst the provision of guidelines ranked in the middle (75.8% scoring ≥ 5/10). 

**Figure 4:**
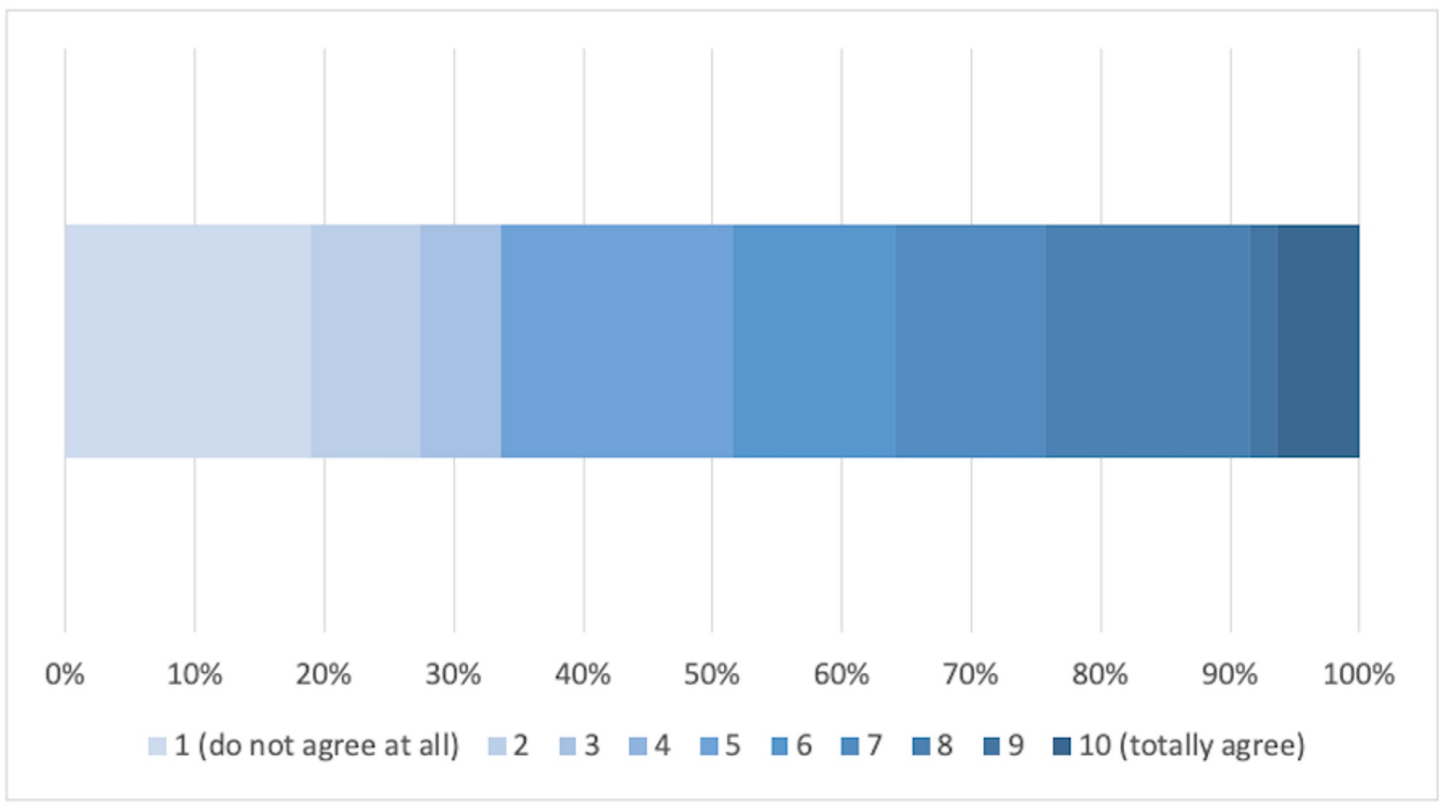
Has the response to COVID-19 led to closer integration within a primary care network (PCN) structure?

As highlighted in Figure 4, 66.3% described greater collaboration with their primary care network (PCN) members as a result of COVID-19 (scoring ≥ 5/10).

Proposals to continue beyond the pandemic

The most common aspirations described by both GPs and PMs were to maintain remote patient consultations (59%) and remote triage (19%). There was also a desire for greater working from home (12%), closer working with the CCG, Local Medical Committee (LMC) and PCN (6%) but at the same time a request for more autonomy and flexibility (5%). 

Other reported positive changes include remote patient monitoring, e.g. blood pressure and peak flow (3%), additional support (3%) and increased respect from governing bodies (2%), simplification of death certification, cremation forms and remote viewing of the deceased (3%), reduced patient expectations (2%) and reduced abuse of NHS services (4%), remote team meetings (2%), fewer tick box exercises such as Care Quality Commission (CQC) visits (2%), longer consultation times (2%), streamlined appraisal and revalidation processes (1 respondent), increased preparation for winters (1 respondent), more regular cleaning (1 respondent) and daily briefings (1 respondent).

Support required to maintain positive changes

The most cited was a request for increased funding and a simplification to the financial structures supporting primary care (21%). 20% felt that significant ongoing patient education is required. Two highlighted examples were education to reduce the medicalisation of social issues and promote self-care as well as teaching patients about how to access the most appropriate resources for their particular issues. 18% demanded improved IT infrastructure, including computers, webcams and better connectivity, as well as a more responsive technical support (10%). 11% called for increased training (especially in IT) and a further 11% sought greater autonomy with a reduced regulatory burden. 13% wanted assurance of greater backing from the regulators (NHS England, CQC, and GMC) in the event of complaints arising directly through the rapid and dramatic rise in the use of telemedicine.

## Discussion

This cross-sectional survey of almost 100 GPs and PMs within LLR found that the COVID-19 pandemic has certainly triggered dramatic changes within primary care. Arguably the most significant was the adoption of teleconsultations, in particular video consults, with largely positive feedback from both patients and GPs. Study findings suggests COVID-19 has substantially accelerated the pace of technological change within NHS primary care with the ‘right’ to digital services by 2024 [6] effectively occurring four years earlier. In addition, practice premises, systems, and processes have also required significant modifications during the pandemic in order to ensure the provision of safe clinical care, e.g. reception screens, one-way patient flow, and greater infection control measures. 

However, despite their ability to introduce such widescale change virtually overnight, it was concerning to find that over 10% of respondents described how the extra strains had placed their practice at risk of closure. Moreover, over half of respondents felt that they were not provided with adequate PPE for their personal safety or that of their staff. Indeed, over a quarter had required absence directly as a result of the pandemic. Perception of the support provided by NHS England and the CCGs was rather mixed, although additional guidelines were broadly welcomed. 

The most requested enduring modifications relate to remote working, universal triage, and remote consultations. However, in order to support such largescale permanent change, respondents felt that a different funding and financial structure is required because GPs should not be penalised for providing less face-to-face access. There is also a need for an improved IT infrastructure because currently, the images are often of unacceptable quality. In addition, there was a call for greater patient education, not only in terms of them being able to use remote IT solutions but also in terms of their expectations because some patients remained insistent on face-to-face meetings. Moreover, whilst there is a clear desire to retain large scale teleconsultations beyond the pandemic, significant apprehension was also expressed about their longer-term safety profile. Doctors wanted greater training in telephone and video consultations as well as a detailed evaluation to ensure that important cues and diagnoses were not being overlooked. The art of history taking is complex and takes many years to master and it was felt important not to lose that in this rush to digitalise. Other doctors described how consulting remotely was also less personal and it was more difficult to portray their empathy. Thus, overall, there was a request for a more supportive regulatory environment whist these new modes of consultation become more widely accepted by our patients and better evaluated by our profession.

This study provides a snapshot of the opinions of primary care professionals within one area of the UK. A larger nationwide study may reveal significant regional differences. Although almost 100 survey responses were received, it is disappointing that the actual response rate was only 7%. This was partly expected because of the shear length of the survey. However, it may also be that the clinical pressures of the pandemic and the need to keep abreast of numerous new guidelines as well providing daily situation reports to the CCG were important deterrents. Nevertheless, what was missing in quantity was appropriately compensated for by the quality of the responses received because most participants were highly experienced and had clearly taken considerable time in providing detailed feedback. A further limitation is that the patient perspective was obtained second hand from NHS professionals, but a direct user survey is proposed. Finally, this is a relatively early canvasing of opinions, but the results may be very different if a follow-up review is conducted in 12 months’ time. 

## Conclusions

Overall, the authors conclude that COVID-19 has placed significant strain upon NHS primary care. However, the pandemic has also substantially accelerated the pace of change with a virtually overnight revolution. The long-term fear amongst many GPs and PMs is that there may be insufficient financial and clinical backing from regulatory bodies to support such rapid and far-reaching changes.

## Figures and Tables

**Figure 5:**
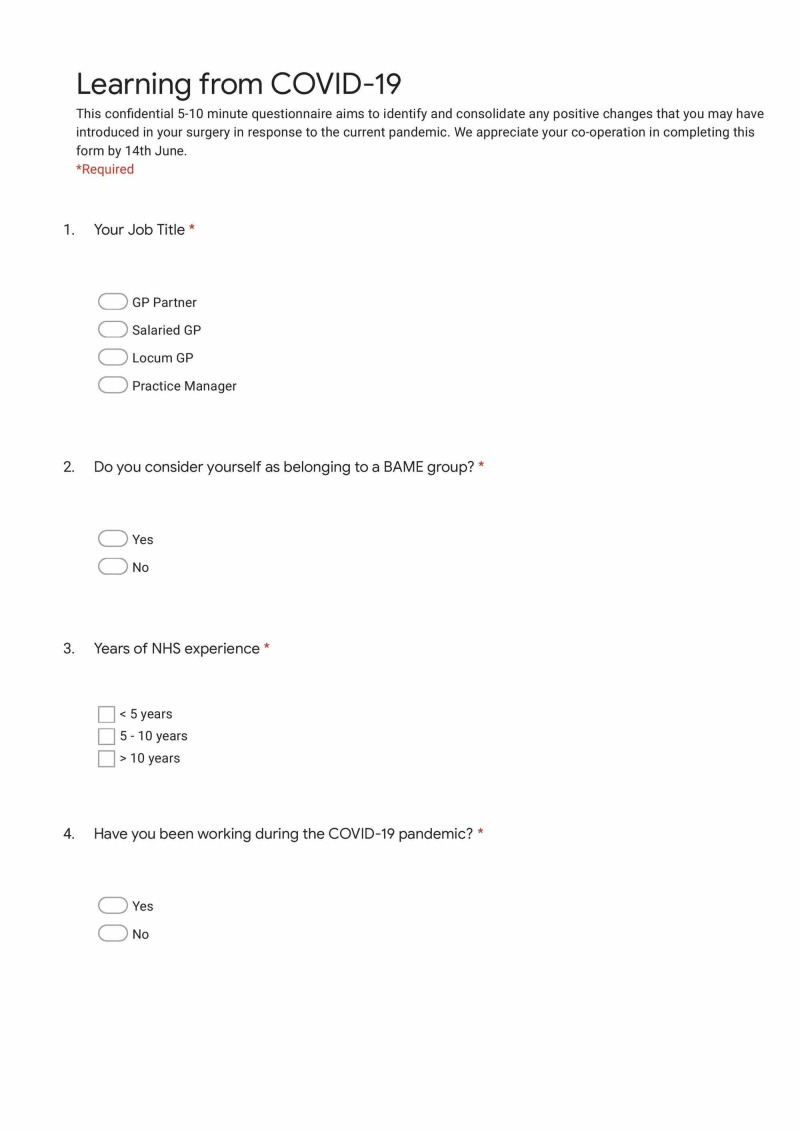
Google Forms Survey Page 1

**Figure 6:**
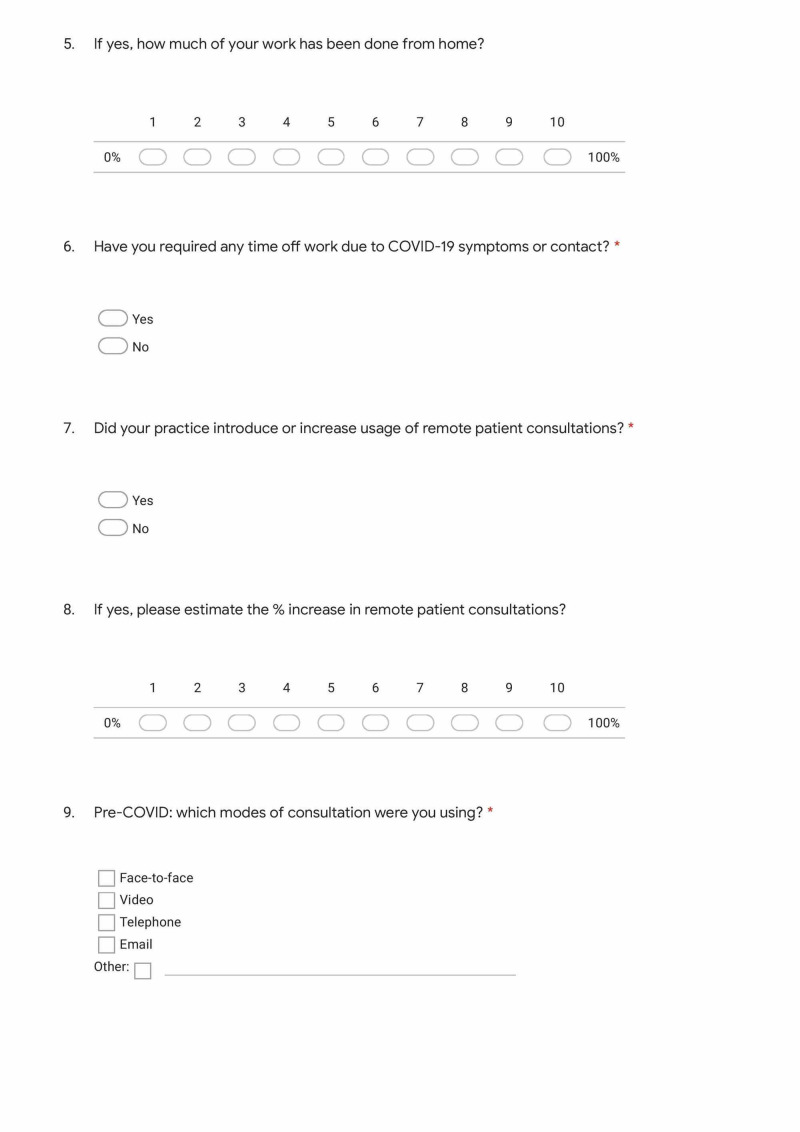
Google Forms Survey Page 2

**Figure 7:**
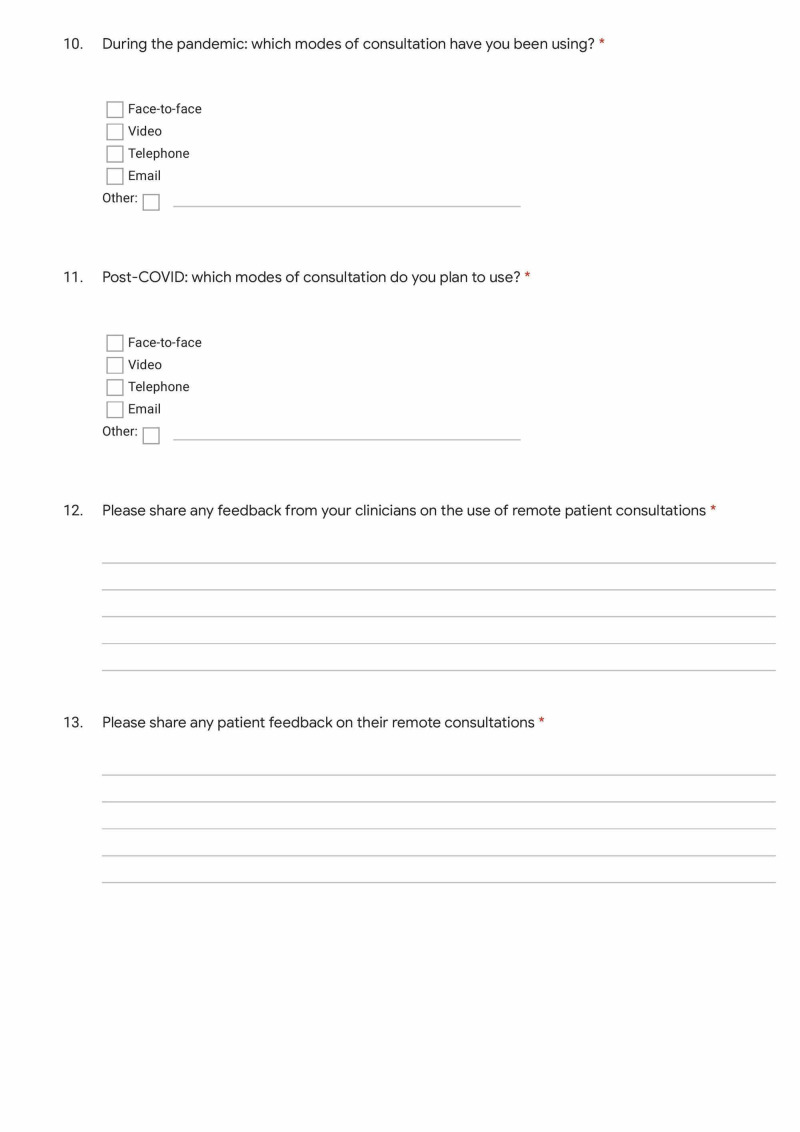
Google Forms Survey Page 3

**Figure 8:**
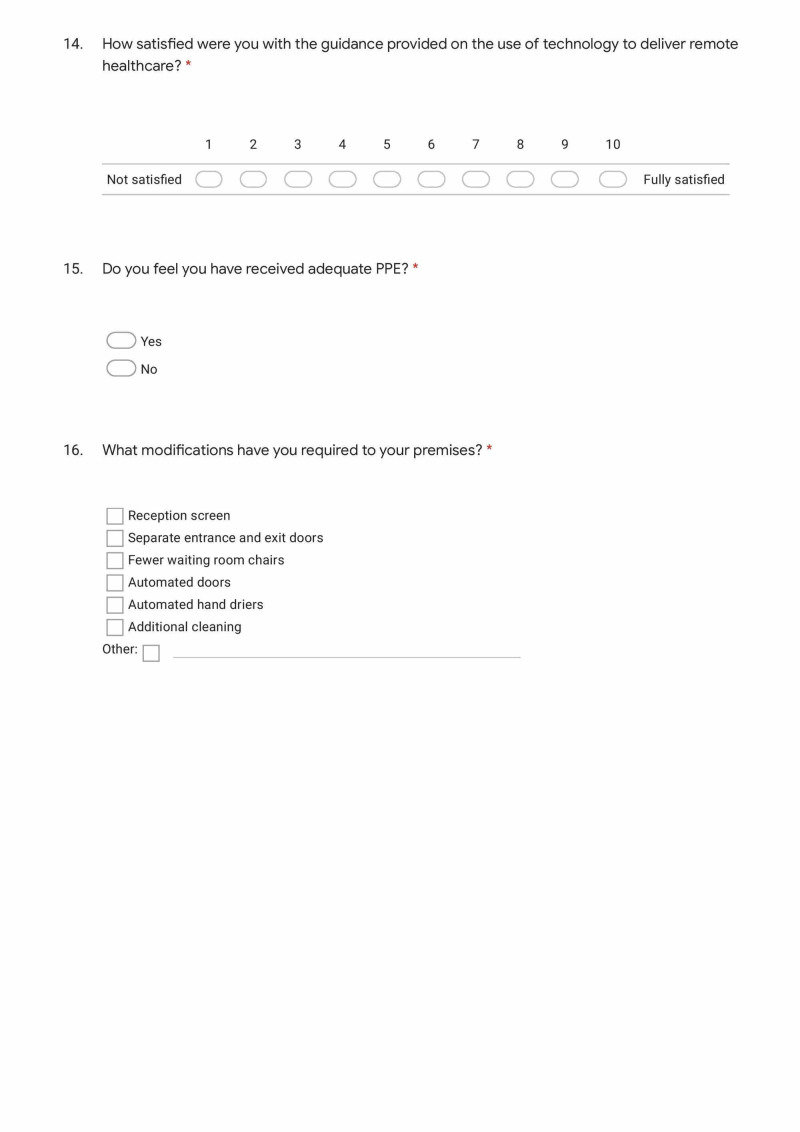
Google Forms Survey Page 4

**Figure 9:**
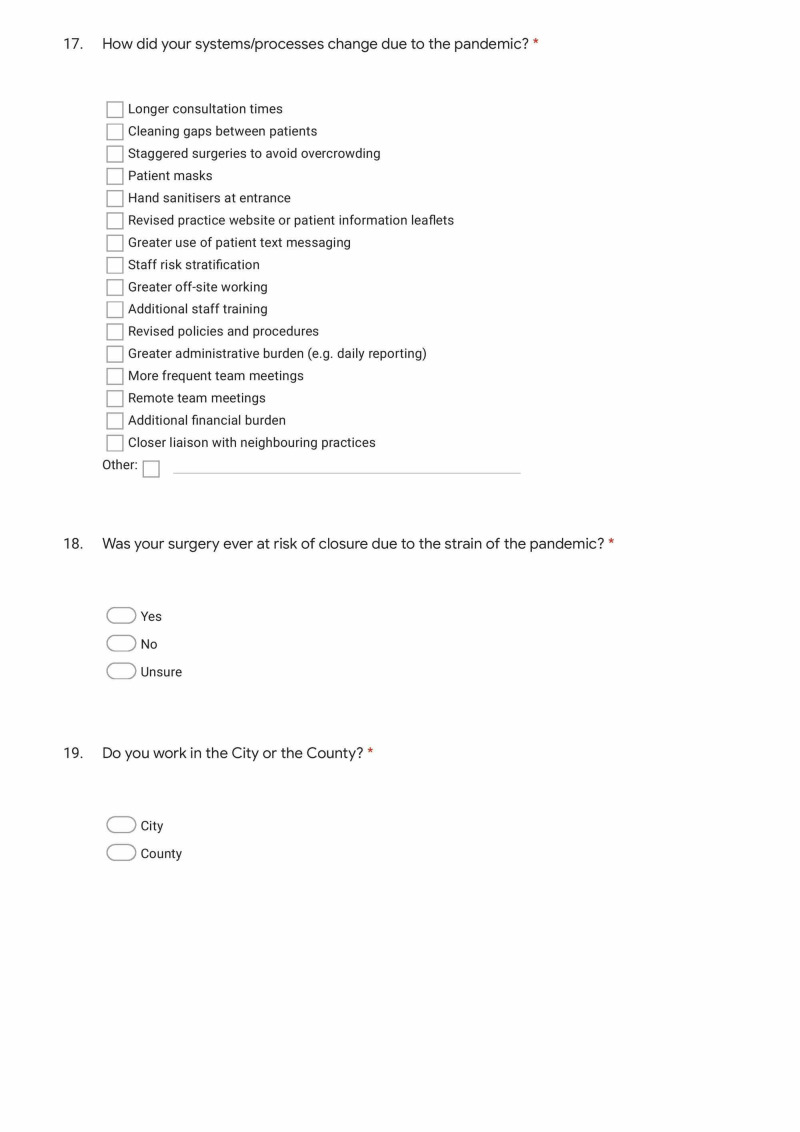
Google Forms Survey Page 5

**Figure 10:**
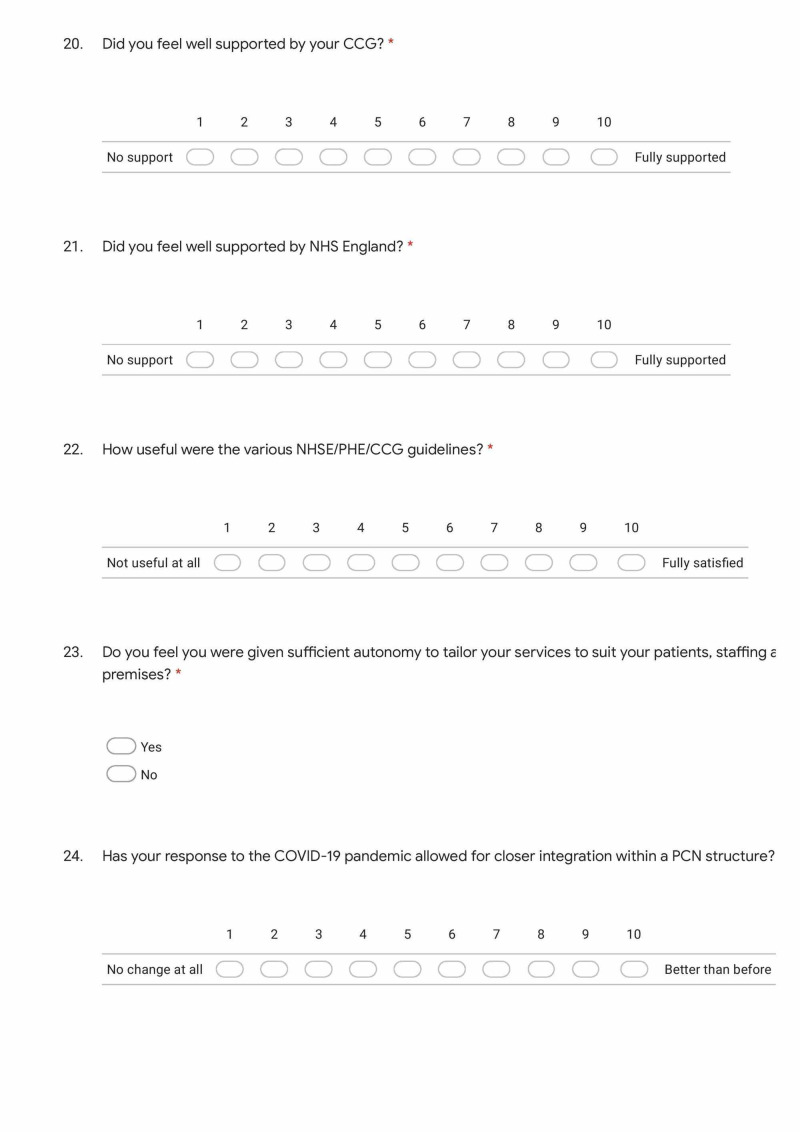
Google Forms Survey Page 6

**Figure 11:**
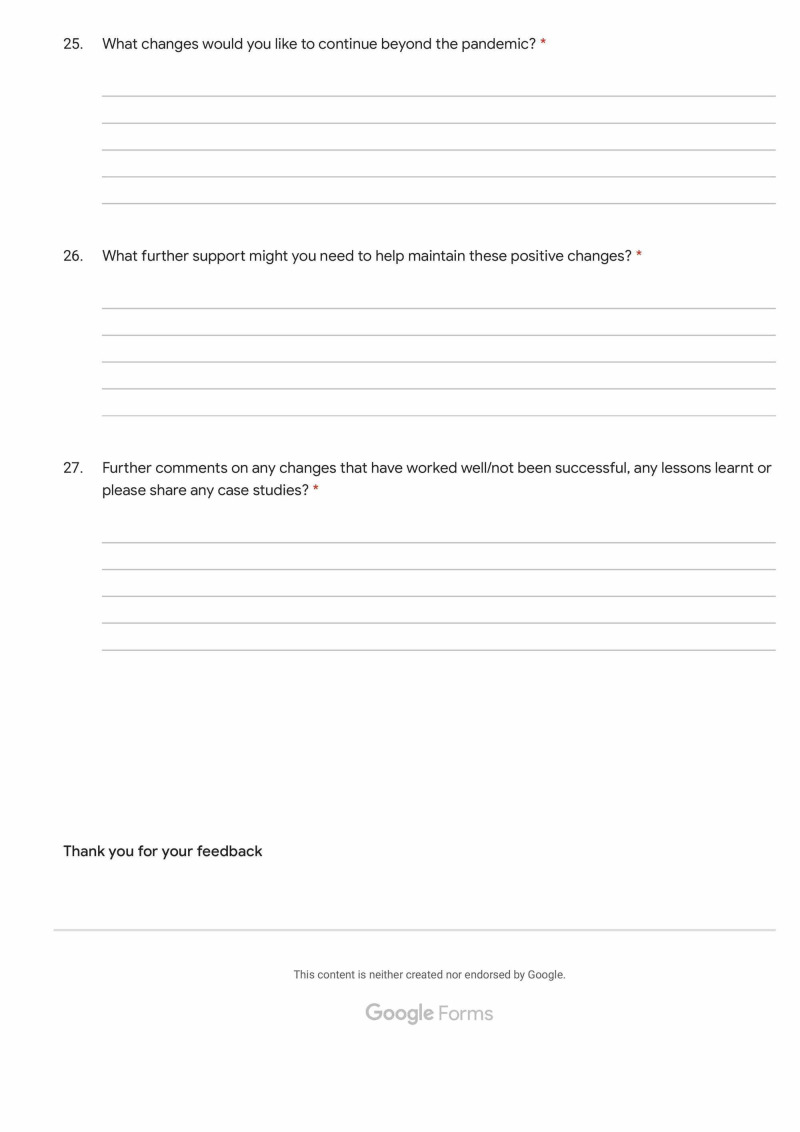
Google Forms Survey Page 7
